# A Low Cost Metal-Free Vascular Access Mini-Port for Artifact Free Imaging and Repeated Injections in Mice

**DOI:** 10.1371/journal.pone.0065939

**Published:** 2013-06-18

**Authors:** Teresa Fiebig, Giovanna Figueiredo, Hanne Boll, Hans Ulrich Kerl, Ingo S. Noelte, Alex Forster, Christoph Groden, Martin Kramer, Marc A. Brockmann

**Affiliations:** 1 Department of Neuroradiology, University Medical Center Mannheim, University of Heidelberg, Mannheim, Germany; 2 Department of Veterinary Clinical Sciences, Small Animal Clinic, Justus-Liebig-University, Giessen, Germany; 3 Department of Diagnostic and Interventional Neuroradiology, University Hospital of the RWTH Aachen, Aachen, Germany; Mayo Clinic College of Medicine, United States of America

## Abstract

**Purpose:**

Small injection ports for mice are increasingly used for drug testing or when administering contrast agents. Commercially available mini-ports are expensive single-use items that cause imaging-artifacts. We developed and tested an artifact-free, low-cost, vascular access mini-port (VAMP) for mice.

**Procedures:**

Leakage testing of the VAMP was conducted with high speed bolus injections of different contrast agents. VAMP-induced artifacts were assessed using a micro-CT and a small animal MRI (9.4T) scanner ex vivo. Repeated contrast administration was performed *in vivo*.

**Results:**

With the VAMP there was no evidence of leakage with repeated punctures, high speed bolus contrast injections, and drawing of blood samples. In contrast to the tested commercially available ports, the VAMP did not cause artifacts with MRI or CT imaging.

**Conclusions:**

The VAMP is an alternative to commercially available mini-ports and has useful applications in animal research involving imaging procedures and contrast agent testing.

## Introduction

Murine models are being used with increasing frequency in a broad range of studies involving the repeated administration of drugs, the use of contrast agents, blood sampling, small animal imaging, as well as the assessment of other physiologic and pathologic parameters.

Experiments with mice frequently require repeated vascular access for e.g. infusion of drugs or contrast agents. Whereas cannulation of the tail vein allows fast and direct intravascular access, this procedure requires training and still it does not yield a 100 percent success rate [1]. To simplify repeated intravascular infusion in mice, small, subcutaneously implantable vascular access ports (also referred to as mini-ports) were developed to provide direct, easy, and repeated access to the murine vascular system [2], [3], [4]. In our own experiments, we tried different commercially available vascular access ports for mice and found numerous drawbacks: i.) all of the ports contained ferromagnetic components that cause susceptibility artifacts in small animal MRI or CT imaging, ii.) some of them were quite large for subcutaneous implantation in mice and/or had a relatively large dead space volume, iii.) some of the ports did not allow the use of a guide wire technique for insertion into the vascular system, and finally, iv.) all ports were quite expensive, with costs ranging between US $50-120 for a single-use item.

For all of the above mentioned drawbacks we developed a vascular access mini-port (VAMP) for mice. The VAMP was designed to offer the following benefits: i.) it does not induce any relevant artifacts in small animal MRI or CT imaging, ii.) has a minimized dead space volume, iii.) can be used in combination with a guide wire during intravascular insertion, iv.) allows drawing of blood from the vascular system, and v.) with material costs of less than US $2 is quite affordable compared to other commercially available products. In this paper we provide a detailed description of the materials required and the process we used to fabricate the VAMP, as well as the use of the port system and the results of different tests performed to ensure general usability in small animal imaging.

## Materials and Methods

### Materials and fabrication of the VAMP

**Figure 1:**
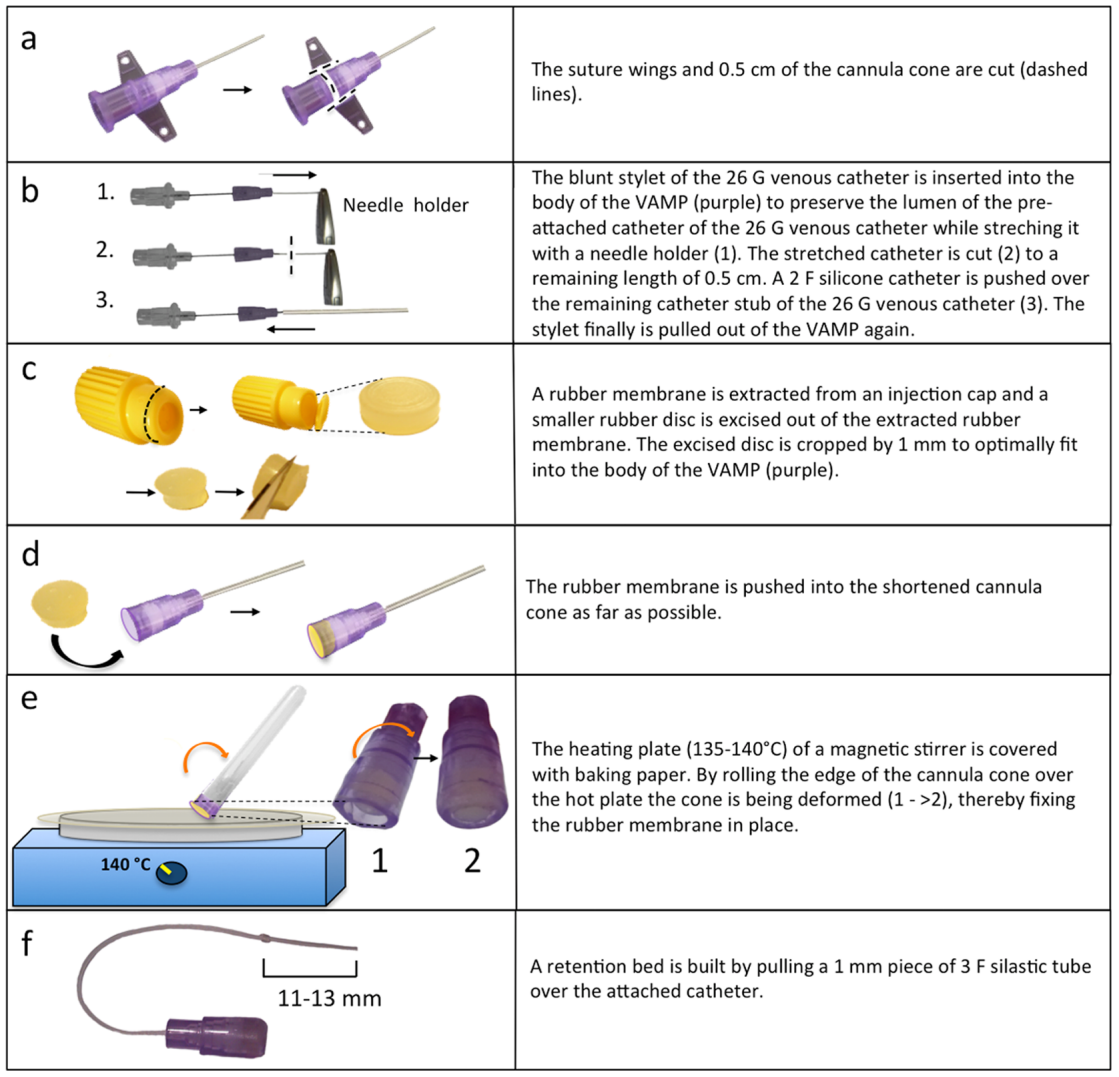
Schematic description on how to manufacture a low-cost metal-free vascular access mini-port (VAMP) for mice.

The VAMP is made from a shortened intravenous cannula cone and a rubber membrane. To prepare the body of the VAMP, the suture wings of a 26 G i.v. cannula (Kliniject; Klinika, Usingen, Germany) are cut with a sharp knife or scalpel, as shown in Fig. 1a. The stylet of the 26 G venous catheter is inserted 1–1.5 cm into the cannula cone to preserve the catheter lumen while longitudinally stretching the catheter with a needle holder, in order to reduce its outer diameter, as shown in Fig. 1b. Next, the stylet is removed again and the stretched catheter cut to a length of 5 mm as illustrated in Fig. 1b. Then, for a second time, the blunt stylet is being used to stabilize the stub of the shortened 26 G catheter and a 5–6 cm long 2 F silicone catheter (Silastic; Dow Corning, Wiesbaden, Germany) is slid over the stub of the shortened, stabilized 26 G catheter (Fig. 1b).

The membrane of the VAMP is made from a rubber membrane that is extracted from a commercially available injection cap (Injection Cap; J. Söllner GmbH, Deggendorf, Germany) as shown in Fig. 1c. To make the rubber membrane fit into the body of the VAMP, a small circular piece is excised from the original membrane using a punch forceps preset to 4.5 mm diameter (E-Top Werkzeuge EDE; Wuppertal, Germany). Using the punch forceps leads to compression of the rubber, which results in a slightly tapered shape of the punched out cylinder. To optimally fit the cylinder into the body of the VAMP, a 1 mm thin slice is cut off the membrane's smaller side (Fig. 1c). The tapered rubber membrane is then pushed, with the smaller side ahead, into the shortened cannula cone as far as possible (Fig. 1d). Next, the membrane needs to be fixed in place within the body of the VAMP. To do so, a heating plate of a magnetic stirrer (RET basic; IKA-Werke GmbH & Co.KG, Staufen, Germany) is covered with baking paper and heated up to 135–140°C. When hot enough, the edge of the cone is bent by slowly rolling it over the hot plate while applying slight pressure to the edge (Fig. 1e). The heat-resistant baking paper (coated with silicone) prevents melting fluorinated ethylene-propylene (FEP) from sticking on the heating plate.

Finally, the 2 F silicone catheter is marked 11–13 mm from the tip to define the maximum insertion depth of the catheter (Fig. 1f), which depends on the weight and size of the animal and (if required) retention beads can be added to the catheter by pulling a 1 mm long piece of a 3 F silicone tubing over the 2 F catheter.

Assembled as described, the VAMP can be sterilized at 121°C for 20 min. or with alcohol.

### Insertion of guide filament

**Figure 2:**
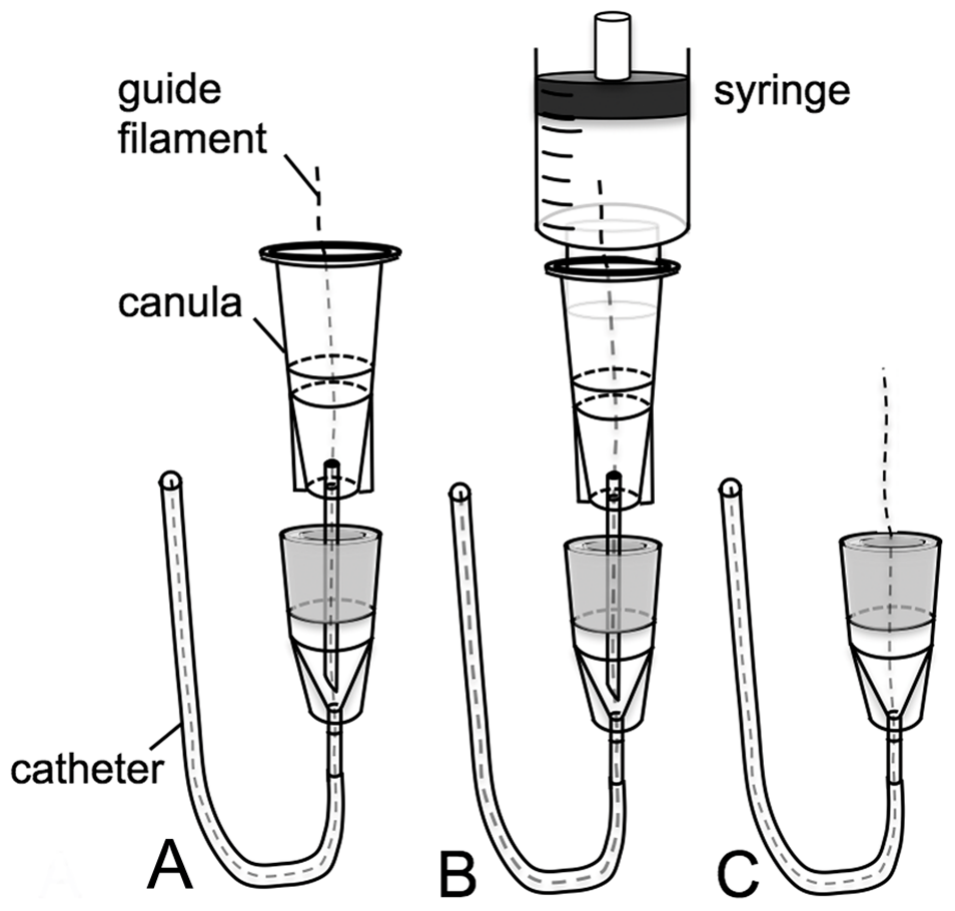
Schematic drawing of insertion of the guide filament into the VAMP. a) A 23 G cannula is inserted into the port and a 0.2–0.25 mm nylon suture is advanced into the catheter tip. b) The port system is flushed with heparinized saline. Note that the proximal end of the nylon suture is advanced into the syringe. c) While flushing the VAMP, the cannula is slowly retracted to provide an air-free lumen of the port and the catheter.

If the 2 F silicone catheter is too floppy to be advanced into a vessel, use of a stabilizing filament within the catheter might be helpful during catheter insertion. For this purpose, a sterile filament (we used a 0.2–0.25 mm Nylon suture) is inserted into the catheter lumen via a 23 G cannula, as shown in Fig. 2. More exactly, a sterile 23 G cannula (BD Microliance; Becton Dickinson, Fraga, Spain) is pushed through the rubber membrane of the sterilized VAMP and a filament is pushed into the catheter. Then the system is flushed with heparinized saline solution while slowly pulling out the cannula to ensure an airfree filling. After successful intravascular insertion of the catheter, the filament can simply be pulled out of the VAMP.

### Surgical implantation of the VAMP

All procedures and housing of the animals were carried out according to the criteria outlined in the “Guide for the Care and Use of Laboratory Animals” prepared by the National Academy of Sciences and published by the National Institutes of Health. All experiments were carried out after receiving local ethics committee approval (Karlsruhe G86/09). Institutional guidelines for animal welfare and experimental conduct were followed.

C57/BL6 mice (21–25 g body weight) were anesthetized by an intraperitoneal injection of ketamine (120 mg/kg body weight, Ketamin 10%; Intervet, Bela-Pharm GmbH&CoKG, Vechta, Germany) and xylazine (16 mg/kg body weight, Rompun 2%; Bayer Vital GmbH, Leverkusen, Germany). When sufficient anesthesia was achieved, the animals were positioned supine on a warmth plate (Medax GmbH; Neumünster, Germany) at 37°C and fixed with tape. Catheterization of the right jugular vein was performed using procedures previously described [5], [6], [7]. Prior to implantation the port and the catheter were flushed with heparinized saline (25 IU/ml).

Postoperatively animals were administered metamizol (Novalgin; Ratiopharm, Ulm, Germany) subcutaneously (200 mg/kg) on the day of the operation and over 3 days via drinking water (200 mg/kg).

In between injections, the VAMP was flushed every other day or every third day with 50 µl of heparinized saline (25 IU/ml). Before flushing, the hair on the back of the animals was clipped and sanitized (Softasept; Braun, Melsungen, Germany) before inserting the needle. The animals underwent DSA via the VAMP once a week for two weeks.

### Repeated puncture and leakage testing at high infusion rates

To conduct leakage testing, the VAMP was punctured 50 times using a new 27 G needle every other puncture. Afterwards, the catheter of the port system was occluded, the port system was punctured for a final time, and pressure was applied by trying to inject sodium chloride solution into the port system using a 2 ml syringe.

Next, we tested the feasibility of injecting different contrast agents with various concentrations (0.9% NaCl, gadoteric acid (0.5 mmol/ml, Dotarem; Guerbet, France), and Iomeprol 150, 200, 300, 400 mg I/ml (Bracco, Konstanz, Germany)) using the VAMP. All fluids were warmed to 37°C before injection to optimize viscosity. Injection of the substances was carried out using an infusion pump (Harvard Apparatus PHD 2000) at the maximally available injection speed (3.4 ml/min) to simulate bolus injection. The time required for injecting 450 µl of each contrast agent into a measuring vessel was recorded.

### *Ex vivo* imaging experiments

To more realistically determine the possible imaging artifacts associated with three commercially available devices (see Table 1) compared to VAMP, we implanted these devices into dead mice and imaged them with both MRI and micro-CT.

**Table 1:** Comparison of the custom-made (VAMP) and the commercially available vascular access ports for mice.

	VAMP (vascular access mini-port)	SoloPort MICRO®	Microport®	Penny MousePort®
**Manufacturer**		Instech Solomon	Braintree Scientific	Access Technologies
**Size**	5×13 mm	4.4×19 mm	3×7×12 mm	6.3×15.8 mm
**Weight**	0.2 g	1.4 g	0.7 g	1.0 g
**Materials**	FEP, Rubber	Steel, Silicone	Steel, Silicone	Steel, Silicone
**Prices**	<2 $	120 $	50 $	80 $
**Repeated punctures**	tested 50×27G	not declared	100×27 G	200×25 G
**Dead space volume**	15 µl	30 µl	10 µl	50 µl

MRI was performed using a dedicated small animal MRI scanner operating at 9.4 Tesla (Bruker BioSpec 94/20; Bruker BioSpin, Ettlingen, Germany) in combination with a volume probe (Bruker BioSpin; T2-TurboRARE, FOV: 3.4×12 cm; slice thickness 2 mm; 21 Slices; matrix: 256×256; voxel size: 0.47×0.13×2.0 mm; TE/TR: 50/3366 ms; flip angle: 180°; number of averages: 1; acquisition time: 1 min 47 s).

Micro-CT was performed as described recently ([8], [9]). Briefly, using an industrial micro-CT (Y.Fox; Yxlon International GmbH, Hamburg, Germany) with a transmission X-ray tube (Yxlon) and a flat panel detector (Varian PaxScan 2520; Varian, Palo Alto, CA, USA), images were continuously acquired at 30 frames per second at a tube voltage of 80 kV and a current of 75 µA within 40 seconds scan time. Image reconstruction was performed using filtered back projection algorithm with a Shepp-Logan filter and a matrix of 512×512×512 voxels, resulting in a voxel spacing of 50×50×60 µm.

### *In vivo* imaging experiments

To provide proof of principle that the newly developed mini-port can be successfully used for repeated injections *in vivo*, the VAMP was implanted in 11 female C57/BL6 mice. As we observed problems with catheter patency in 2 and with post-operative recovery in 2 out of the 11 animals, DSA was performed in the remaining 7 mice using an industrial micro-CT as described recently [10], [11]. Briefly, after positioning the anaesthetized mouse (Forene; Abbot, Wiesbaden, Germany), in a cradle under the X-ray source, gain and offset calibration were performed. After implantation of the VAMP, all mice underwent DSA of the cardiopulmonary vessels. One week later and again two weeks later, three mice underwent cerebral DSA and four mice underwent DSA of the cardiopulmonary vessels. DSA was performed by infusion of a pre-warmed (37°C) contrast agent bolus (Iomeprol 300; target volume 50–100 µl, infusion rate 3.4 ml/min) into the jugular vein, next to the right atrium. Contrast agent application was performed by infusing contrast agent into the VAMP with a catheter, attached to a 1 ml syringe. The syringe was placed into the remotely controlled infusion pump. DSA projections were acquired with 30 frames per second, using a detector pixel matrix of 944×704, a tube voltage of 80 kV, and a 75 µA current. Spatial resolution of DSA ranged between 18×18 µm and 21×21 µm. Acquisition time was manually determined starting few milliseconds before contrast agent application and stopped when contrast agent entered the cerebral veins.

## Results

### Repeated puncture and leakage testing at high infusion rates

Repeated puncture (50 times) using a 27 G needle (new needle every other puncture) with terminal high pressure injection of sodium chloride solution into the VAMP did not result in any leakage through the punctured rubber membrane itself, nor between the membrane and the body of the port.

**Figure 3:**
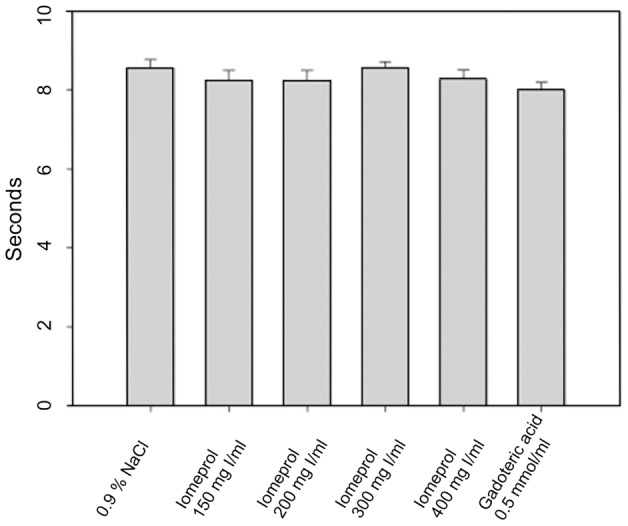
Times required for injection (3.4 ml/min) of 450 µl of different pre-warmed (37°C) contrast agents via the self constructed vascular access mini-port. Despite different concentrations (and thus viscosities) of the contrast agents, no relevant delay of infusion speed compared to a physiological saline solution was observed and no leakage from the VAMP was detected.

Using the maximum injection speed of our injection pump (3.4 ml/min), we found that despite differences in contrast agent concentration and viscosity, there were no notable differences in time until injection of a bolus of 450 µl of contrast agent, or physiological sodium chloride solution through the attached 2 F catheter (5–6 cm length) (Fig. 3).

Interestingly, during our experiments the silicone membrane of the SoloPort MICRO (Instech Solomon) released from the metal housing of the port, resulting in loss of fluid to the outside of the port system. This problem was reproducible in all three tested SoloPorts. The reason for this finding was most likely related to the incompatibility of the system with increased injection rates due to insufficient fixation of the membrane. We did not evaluate the maximally tolerable injection speed for this or the other commercially available systems, as this was not the main goal of our study.

### Animal imaging experiments

**Figure 4:**
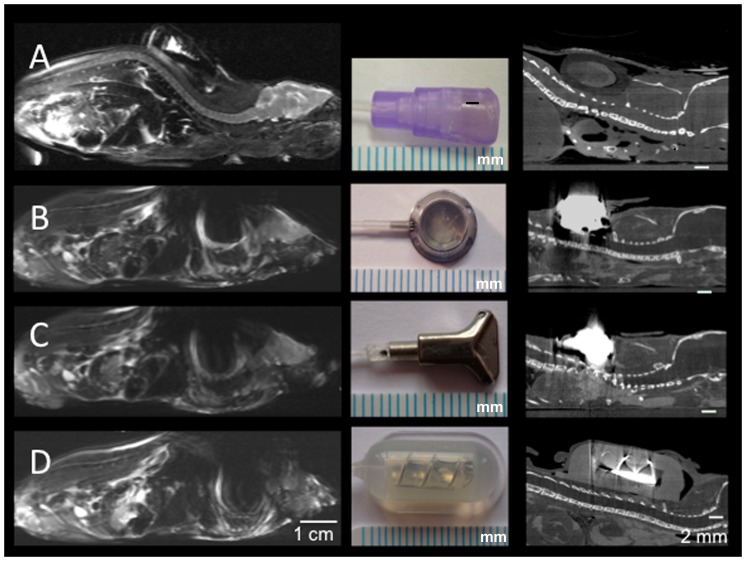
Assessment of port-induced artifacts in mice using a high field strength MRI (dedicated small animal scanner at 9.4 T; T2-weighted sequence) and micro-CT. Whereas the custom-made vascular access mini-port (A) does not cause any relevant artifacts in MRI or micro-CT imaging, all commercially available mini-ports (B: SoloPort MICRO, C: Microport, D: Penny MousePort) cause strong susceptibility artifacts at 9.4 T that hamper proper imaging of mice. In micro-CT, artifacts of the commercially available mini-ports only interfere with thoracic imaging (or other regions, depending on the site of port implantation). Scale below the photographs: 1 mm.

To compare and estimate the degree of port-related artifacts at high field-strengths with small animal scanners, sacrificed mice with subcutaneously implanted ports underwent high resolution MRI at 9.4 T. Whereas all commercially available metal-containing ports caused extensive susceptibility artifacts, making examination of the head and thorax as well as most of the abdominal organs almost impossible, the VAMP caused no relevant artifacts (Fig. 4). Interestingly, the port causing the strongest susceptibility artifacts in MRI (Penny MousePort) produced weaker beam hardening artifacts. This is explained by the fact that the port's housing is not made of solid metal (i.e. less beam hardening), but that the metal used is configured like a spiral coil, causing stronger susceptibility artifacts in MRI.

**Figure 5:**
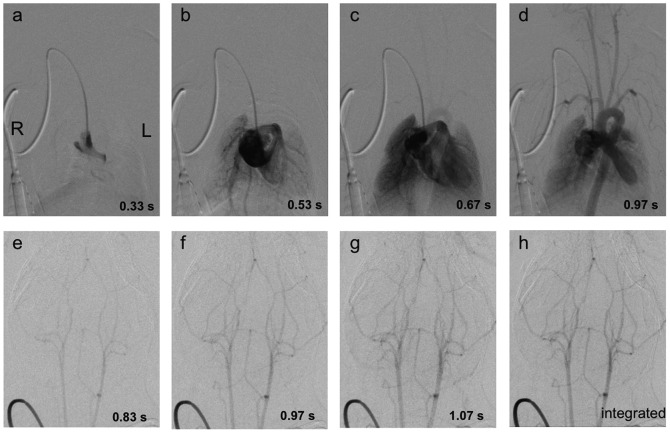
Digital subtraction angiography of a C57/BL6 mouse was performed during injection of 100 µl Iomeprol 300 using the VAMP. Sequential enhancement of the right atrium (a), the right ventricle and lung arteries (b), the lung parenchyma (c), the left ventricle, the aorta and supra-aortic arteries (d) is shown. A digital subtraction angiography of the cerebral vessels is shown the second row. Images (e)–(g) show the sequential enhancement of the cerebral arteries within one second. Integration of images was performed to improve image quality (h).

Using the VAMP for repeated contrast agent administration, DSA was performed weekly over two weeks, as shown in Fig. 5. We observed problems with catheter patency in 2 and with post-operative recovery in 2 out of 11 animals. The remaining 7 animals were used for the underlying studies. Macroscopic post-mortem examinations of the implantation area showed normal tissue reactions after port implantation (fibrin) and in one case a small subcutaneous haematoma, but no other relevant changes.

## Discussion

Administration of contrast agent is frequently used in pre-clinical imaging [12], [13]. While several approaches for contrast agent infusion like canulation of the lateral tail vein [14] and carotid artery [11] are used, we preferred the cannulation of the jugular vein in order to gain the required central venous access.

Chronic catheterization systems used with freely moving small animals have been developed to provide easy and repeated access to the murine vascular system: these include tether-systems [5], [6], [7], [15], [16] and small subcutaneously implantable injection ports [2], [16], [17], [18]. Tether-systems to some degree mean a restrainment for the animal and require restrainment-associated single housing of the animals. Furthermore, tether-systems suffer from relatively large dead space volumes ranging between 24 and 80 µl [5], [19]. The main advantage of tether-systems is that repeated injections can be performed without touching the animal. Subcutaneously implantable small animal injection ports provide an alternative to tether-systems: they have a smaller dead space volume and allow the freely moving animals to be kept in groups [7], [16], [20]. On the other hand, manipulation of the animal is required in order to inject substances.

Searching for an injection port system matching our needs when performing cerebrovascular imaging in mice, we found several commercially available port systems from different manufacturers. To circumvent the disadvantages of the available ports we decided to develop a small, metal-free vascular-access mini-port, that can be used with a guide wire or filament to simplify intravascular advancement of the catheter for untrained staff. Although using a stiff polyethylene tube makes insertion of the catheter easier without a guide wire, it increases the risk of vessel perforation. More information on the pros and cons of various catheter materials and their usability and maintenance in rodents has been published elsewhere [16], [21].

For perfusion measurements or imaging of the arterial system a possibly short and concentrated bolus of contrast agent is advantageous. Thus we considered a central venous catheter to be advantageous compared to tail vein injections. To determine the perfect insertion depth of the catheter, we referred to several publications [5], [6], [19] where the authors suggested insertion depths between 11 and 13.0 mm depending on the animal's weight. Correct placement of the catheter has been verified by monitoring the appearance or disappearance of the heartbeat against the catheter [19] or by a blood aspiration test [6]. If available, we found that x-ray imaging is a good way to confirm the correct insertion depth and location of the catheter.

Catheter material, diameter, and the use of different lock-solutions and various flush intervals have been discussed in the literature. While the chosen catheter diameter primarily depends on the size of the vessel used for catheter implantation and the required injection speed, a smaller diameter of the catheter has been associated with a greater probability of remaining patent [22]. Thomsen et al. [23] recommended flushing of the catheter every day. Chistyakova et al. [24] also recommended daily flushing, however, they also described the use of a lock solution (heparin and dextrose) to lengthen the flush intervals. Different lock solutions have been investigated by Luo et al. [25] in rats, with heparinized glycerol showing the best results to help maintain catheter patency up to four weeks. The efficacy of heparin coated catheters has been discussed controversially [26], [27].

## Conclusions

As the VAMP virtually eliminates port-related artifacts, this vascular access mini-port for mice can be helpful for all kinds of animal studies requiring repeated intravascular injections or imaging procedures. In addition, the fabrication of the VAMP is considerably less expensive, making it a viable and cost effective alternative to commercially available mini-ports.
